# Short peptide epitope design from hantaviruses causing HFRS

**DOI:** 10.6026/97320630013231

**Published:** 2017-07-31

**Authors:** Sathish Sankar, Mageshbabu Ramamurthy, Balaji Nandagopal, Gopalan Sridharan

**Affiliations:** 1 Sri Sakthi Amma Institute of Biomedical Research, Sri Narayani Hospital and Research Centre, Sripuram, Vellore 632 055, Tamil Nadu, India

**Keywords:** Short peptide, epitope design, hantaviruses, HFRS

## Abstract

Several genotypes of the hantavirus cause hemorrhagic fever with renal syndrome (HFRS) and is an important public health problem worldwide. There is now growing interest to develop subunit vaccines especially focused to elicit cytotoxic T lymphocyte responses which are important against viral infection. We identified candidate T-cell epitopes that bind to Class I HLA supertypes towards identifying potential subunit vaccine entity. These epitopes are conserved in all 5 hantavirus genotypes of HFRS (Hantaan, Dobrava- Belgrade, Seoul, Gou virus and Amur). The epitopes identified from S and M segment genomes were analyzed for human proteasome cleavage, transporter associated antigen processing (TAP) efficiency and antigenicity using bioinformatic approaches. The epitope MRNTIMASK which had the two characteristics of high proteasomal cleavage score and TAP score, also had high antigenicity score. Our results indicate that this epitope from the nucleocapsid protein may be considered the most favorable moiety for the development of subunit peptide vaccine.

## Background

Hemorrhagic fever with renal syndrome (HFRS) is a highly fatal disease caused by different genotypes of Hantaviruses (family Bunyaviridae). The disease process includes acute shock, vascular leakage, and acute kidney failure [1]. The genotypes that cause HFRS are Hantaan, Dobrava-Belgrade, Seoul, Gou and Amur. This disease poses a significant public health burden in Asian countries. A recent report from China indicates that the incidence rate of HFRS was about 1.96 cases/100,000 persons [2]. Effective antiviral treatment is not available and vaccine for the control of hantavirus infection is still under development [3,4]. There is now interest for developing T-cell epitope based vaccines to protect against important viral infections [5,6, 7]. Recent bioinformatic techniques including immunology-focused resources and software highly complement in designing candidate vaccine [8]. These methods enable the systematic identification of genome-wide expressed proteins bearing T-cell epitopes. This is achieved by incorporating epitope prediction tools in analyzing large numbers of viral amino acid sequences. Proteasome is a key factor in the degradation of cytosolic proteins in which the C-terminal ligand of Class I HLA is selected [9]. The effective epitope antigen should have a sequence that survives the proteome and can be transported by Transporter associated with antigen processing (TAP) molecule. The TAP molecule is a transporter, associated with the MHC Class I restricted antigen processing [10]. The processing of precursor proteins into MHC Class I ligands by the proteasome, other proteases and the TAP transporter has been studied extensively. The data from these studies have been used to construct successful processing-predictors [11]. The candidate Tcell epitope vaccine that has binding affinity with more than one major histocompatibility complex (MHC) allele also known as promiscuous binders are considered most favorable because it is presented by MHC antigens of major human population groups. The human leukocyte antigen (HLA) supertype is a set of HLA alleles with overlapping peptide binding specificities. The alleles in the given HLA supertype often present the same epitope [12]. This could have considerable implications for T-cell epitopebased vaccination strategies. Previously, Yoshimatsu and Arikawa [13] reported that the epitopes of nucleocapsid protein of hantaviruses were immunodominant. They demonstrated this feature using observation on dynamic differences in the properties with monoclonal and polyclonal antibodies binding to epitopes in Yeast two-hybrid assay and competitive ELISA.

We analyzed HFRS causing hantavirus genomes to design a candidate T-cell epitope vaccine using immunoinformatics strategies. The peptide epitopes that bind to Class I HLA supertypes and stimulate T-cell immune responses are predicted by the NetMHCpan software. We aimed to identify candidate Tcell epitope(s) conserved in all 5 hantavirus genotypes (Hantaan, Dobrava-Belgrade, Seoul, Gou and Amur) causing HFRS. The epitopes that bind to Class I HLA supertypes that is also cleaved only at the flanking regions by human proteasomes, binding to TAP efficiently was identified by the programs MAPPP and TAPpred respectively. This study enabled the prediction of MHC Class I binding T- cell Epitopes from hantaviruses towards development of a designer peptide vaccine.

## Methodology

### Retrieval of sequences

**Figure 1:**
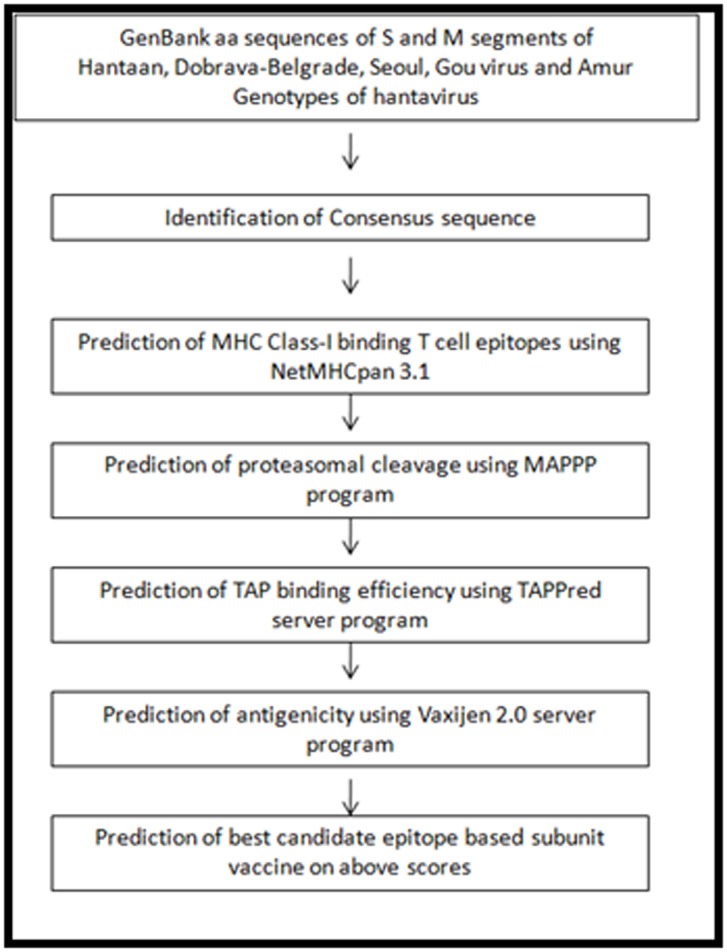
Flowchart indicating the study design

All available complete S and M segment amino acid (aa) sequences of genotypes Hantaan virus (n=152), Dobrava- Belgrade virus (n=44), Seoul virus (n=102), Gou virus (n=36) and Amur virus (n=9) that causes hemorragic fever with renal syndrome (HFRS) were retrieved from GenBank database [14] as of October 2016. The S segment consensus amino acid sequence was 429 and M segment consensus amino acid sequence was 1135 in length. The study design flow chart is shown in Figure 1.

A consensus aa sequence for each genotype was identified using CLC sequence Viewer 7 program [15]. The program identifies the consensus sequence based on most frequent residues found at each position in the sequence alignment. The consensus sequence was used for further analysis to identify T-cell epitopes.

### Prediction of MHC Class I binding T cell epitopes

We selected the Class I MHC supertype representative alleles (n=12) for the prediction of specific T-cell epitopes as available in the NetMHCpan 3.1 online prediction server program [16]. The epitopes of 9-mer length (nonamer) was derived with the default threshold for strong binding and weak binding in terms of percent rank. Strong binders alone were selected and used for further analysis as shown below:

### Prediction of proteasomal cleavage

MHC-I Antigenic Peptide Processing Prediction (MAPPP) program [17] was used to predict proteasomal cleavage. The program generates a probability for the cleavage of each possible peptide from a protein by the proteasome in the cell and the probability is based on a statistic-empirical method. The algorithms in the program were earlier implemented in FRAGPREDICT. Minimum possibility for cleavage after a single residue and for cleavage of a fragment was set to a default value of 0.5.

### Prediction of TAP efficiency

The TAPPred server program [18] was used to predict the candidate epitope(s) based on the predicted processing of the peptide(s) in vivo, the transporter of antigenic peptides (TAP) proteins' transport efficiency. The prediction approach used in this study was the cascade support vector machines (SVM), a prediction that is based on the sequence and features of amino acids and their properties.

### Prediction of antigenicity

The identified epitope(s) were used to predict whole protein antigenicity (protective antigen) using Vaxijen 2.0 server program with a threshold limit of 0.5 [19]. The threshold values of the highest accuracy of more than 0.5 were considered probable antigens and were selected for further analysis.

## Results

The amino acid sequence of S segment and M segment were analyzed for the determination of their respective T-cell epitopes. The 12 HLA supertype representatives analyzed in this study include HLA-A*01:01, HLA-A*02:01, HLA-A*03:01, HLAA* 24:02, HLA-A*26:01, HLA-B*07:02, HLA-B*08:01, HLA-B*27:05, HLA-B*39:01, HLA-B*40:01, HLA-B*58:01, and HLA-B*15:01. Class I MHC binding T-cell epitopes binding to each of the above HLAs were predicted individually for the S and M segment amino acid sequence of the five Hantavirus genotypes causing HFRS. The default rank threshold for strong binding peptides and weak binding peptides were set at 0.500 and 2.000 respectively. The epitopes that had high binding levels (strong binders) were selected for proteasomal and TAP evaluation.

In the S segment, the T-cell epitope prediction online server program identified 421 epitopes for each HLA supertype. Among these, the epitopes that are "strong binders" ranged from one to six. HLA-B*07:02 and HLA-B*08:01 showed 3 shared epitopes in Amur virus, and other viruses showed two shared epitopes. However, in M segment, no common epitopes were seen between the two HLA predicted epitopes. In the S segment amino acid analysis, Dobrava-Belgrade, Seoul and Gou viruses did not show any strong binding epitope for HLA-A*01:01. Four epitopes were commonly present in all the five genotypes (Table 1). The chosen epitopes, MRNTIMASK binding to HLA-B*27:05 and WGSGVGFTL binding to HLA-B*39:01 were checked against available aa sequences in the Repository for Epitope datasets (http://ailab.ist.psu.edu/red/mhci.html) and not found. This database possesses a large set of 48,828 quantitative peptidebinding affinity measurements relating to 48 different mouse, human, macaque, and chimpanzee MHC class I alleles. So, our epitopes are to be considered novel in the context of HFRS genotype-derived immunogens.

In the S segment, further screening based on proteasome cleavage resulted in one epitope with a score above the set threshold. The other three epitopes were not predicted by MAPPP because the proteasomal protein degradation was indicated within the epitope; they however had intermediate or high TAPpred score. The epitope MRNTIMASK which had the two characteristics of high proteasomal cleavage score and TAPpred score, also had high antigenicity score (Table 1).

In the M segment amino acid analysis, a total of 1127 epitopes were identified for each HLA supertype. The "strong binders" ranged from six to sixteen. Among the 5 genotypes, six epitopes were commonly present, of which only one (WGSGVGFTL) had proteasomal cleavage site at the flanking regions of the peptide with good antigenicity score. However, the epitope had low TAP score.

## Discussion

Many genotypes of hantaviruses have been reported to cause HFRS. New strains of hantaviruses are now reported by molecular testing and/or genome sequencing as distinct genotypes [20]. The hantaviruses are identified either directly from human samples or from the rodent hosts trapped in areas where clinical cases are recognized [21,22]. The five genotypes (Hantaan, Dobrava-Belgrade, Seoul, Gou viruses and Amur) were selected for this study because only these viruses have been identified from human subjects with the illness till date. Our study included all available full length sequences of S and M segments retrieved from the GenBank repository. A consensus sequence was prepared by the CLC sequence viewer program based on the most frequent residues found at each position in the sequence alignment.

Promiscuous presentation of T-cell epitopes by multiple HLAs is particularly important in the generation of protective immune response. Hence, this feature has to be borne in mind for vaccine development. A vaccine prepared based on this principle “designer vaccine” will be effective in a high proportion of the human population [8]. Human Class I HLAs (HLA-A, HLA-B, HLA-C) are highly polymorphic and play an important role in the presentation of antigenic peptides to the TCR expressed by CTLs [23]. Ethnic variation in HLA allele frequencies among populations is an important factor to be considered while understanding vaccine-mediated immunity. There is an increased interest to develop human vaccines against important viral infections based on the HLA diversity of the particular population [24]. Vaccine against Hantavirus, an important etiological agent of HFRS has been reported [25]. In this study the analysis however was restricted to a specific HLA (A*02) using mouse model. In the case of Hantaviruses, multiple genotypes circulate in different geographical areas. Hence, there is a requirement for a universal “designer peptide vaccine” that induce specific immunity to all genotypes. Therefore, we included all the five important genotypes that cause HFRS to design a HLA supertype alleles compatible vaccine.

Promiscuously recognized T cell epitopes have been identified from a variety of different disease targets, including measles mumps-rubella, SARS, EBV, HIV, Kaposi' Sarcoma Associated Human Herpesvirus, HCV, HBV, HPV, influenza, P. falciparum, LCMV, Lassa virus, F. tularensis, vaccinia, and also cancer antigens [26]. The development of T-cell epitope vaccine needs further evaluation [27].

Peptide vaccines reported previously have looked at antigenic epitopes and tested its effectiveness. However, in our study, a unique approach was undertaken to design a peptide vaccine based on the concept of matching the epitopes to the prevalent HLA haplotypes in a given population.

Although more than 11,000 HLA alleles have been identified so far, most HLA molecules can be clustered into supertypes based on their overlapping peptide-binding specificities or the residue composition at their peptide-binding sites [28]. Sidney et al. [29] documented HLA-A*0201 binding peptides also cross-bind with other A2-supertype molecules thus indicating a peptide that binds to a HLA molecule with high affinity can also bind to multiple HLAs within the same supertype. Such promiscuous presentation of T-cell epitopes that are HLA supertype-specific are ideal for the development of vaccine particularly for the population whose HLA diversity has not been well characterized and reported. Our approach will thus help develop peptide vaccines targeted at specific infectious agents for use in different population groups. The program NetMHCpan 3.1. is integrated with options to select HLA supertype representatives in addition to all available HLA alleles. We chose supertype representatives because allele-specific data are not well described for the much diverse Asian population. Candidate vaccine peptides with promiscuous recognition of antigens to HLA supertypes have been reported previously for many different pathogens [30, 31][32]. The efficacy of such supertype-based method was examined in two matrix-based methods and one machine learning method for 20 alleles in HLA supertypes A1, A2, A3, A24, B44 and B7 and shown to remarkably improve the prediction of HLA-binding peptides [33].

The Hantavirus nucleoprotein plays an important structural and functional role in host pathogen interactions. The protein forms stable trimers, and are required for specific binding to the RNA panhandle that arises from base pairing of the terminal sequence [34]. Similar viral nucleoproteins of Influenza A virus are the primary targets for vaccine design [35]. The programs TAPPred and MAPPP for TAP and proteasome cleavage respectively select out sequences which survive proteolytic cleavage and thus bind to TAP for translocation to the pockets of MHC class-I with strong affinity of binding for 9-mer peptide [36].

In this study on S segment analysis, among the epitopes identified for the five genotypes, only one peptide MRNTIMASK had high proteasomal cleavage, TAP efficiency, and antigenicity score predicted to bind to HLA-B*27:05. The HLA-B*27 family with at least 100 different alleles, are widely distributed in the human population. These HLA-B*27 molecules are reported to be an important host factor associated with virus clearance. This HLA termed as "protective molecule" showed to reduce the ability of HCV to escape the cytotoxic T-cell response of the host [37]. A similar approach was carried out by Shehzadi et al. [38] in the selection of epitope-based vaccine which targeted HCV genotype 1 using complete genome sequences based on HLA binding prediction score alone.

The peptide identified in this study was shown to bind to HLA supertype HLA B27:05. HLA-B27 has a high degree of genetic polymorphism and is distributed throughout the world. HLAB27 supertype allelic representation and its global distribution with country-wise predominance including South Asia has been reported [26, 39] for whom the vaccine could be targeted.

The HLA supertypes and their polymorphic variants are identifiable and prevalent in a given population. It is inferred that the variants do not behave differently in terms of epitope presentation. The HLA supertypes that the program took into consideration for analysis of T-cell epitope presentation for HFRS strains were 12. It could be surmised that these 12 supertypes would cover a large proportion of the population.

Significant number of HLA class I molecules are classified into a few supertypes. This is characterized by overlapping peptidebinding repertoires. The peptide binding to consensus B- and Fpocket structures are documented and cross-binding peptides are distinguished by specific T cells [40].

The peptide reported in the manuscript has been selected based on analytical results from MHC binding, TAP efficiency and proteasome cleavage. The chosen peptide has also been shown to be specific to HFRS causing Hantaviruses in BLAST analysis. Therefore, this peptide is considered suitable for peptide-based vaccine development against HFRS causing Hantaviruses. Previously, single peptide vaccine has been shown to be effective and highly protective against experimental Salmonella infection [41].

Multiple antigen presenting (multibranched) peptide vaccine encounters the difficulty of purification to homogeneity and characterization of the final products [42]. Furthermore, if they are not MHC matched, the immune response outcome will be unpredictable. Currently, there is an approach to develop peptide vaccine using chimeric proteins from mosquito saliva as a vaccine. The thinking is that one could prevent the efficiency of mosquitoes' blood meal thereby preventing successful viral inoculation into the host [43].

Previously, overlapping CD8+ CTL epitopes from Hantaan virus nucleocapsid protein was shown to elicit IFN-γ production in vitro. This epitope was found to be restricted by HLA alleles (A11, A24, and B7) [44]. Though, MHC-peptide binding is a crucial part in mounting of a specific immune response, several biochemical events occur within the cell before antigen presentation to CTLs. Presentation on professional or nonprofessional antigen presenting cells (APCs) depends on the proteasome, a large cytosomal protease complex for generation of antigenic peptides. For further processing, the peptides must enter the endoplasmic reticulum by active transport mediated by the transporter associated with antigen processing (TAP) and presented on MHC class I molecules on the surface of either infected cells or APCs [9]. Hence, in our study, for more meaningful inference, T-cell epitopes that bind to Class-I MHC molecules and survive proteasomal cleavage with transport efficiency by TAP proteins was investigated. In addition, prediction of protective viral antigens was made using Vaxijen model. The model is reported to have prediction accuracy up to 89% [45].

B-cell epitopes have been reported for the N protein coded by S segment of Hantaviruses causing HFRS [46]. However, immunodominant T cell responses are central in the control of any acute virus infection. A study reported weakening of IFN-γ- producing T cell response in patients with severe HFRS at the early stage of infection [47]. The study suggested that insufficient T cell response to the immunodominant epitopes might play a role in influencing the severity of HTNV infection. Hence, a wellchosen T-cell epitope vaccine administered as prophylaxis will alter the course of infection in favor of the vaccinated host.

In hantavirus infections of experimental animals there is clear evidence of both B- and T-cell responses. Serum antibodies could prevent viremic spread and T-cell responses (cytotoxic T cells) could eliminate infected cells [48]. Furthermore, in human HFRS, both the Th1 and ThGzmB+ (CD4 CTL) cell responses against HTNV glycoprotein are shown in the CTL response. The CD8 CTL lyse the infected host cell by perforin mechanism, whereas, CD4 CTL cause apoptosis through Granzyme B mechanism [49].

A DNA vaccine was developed from Andes virus envelope glycoproteins coded by the M segment that causes HCPS. The vaccinated geese developed high-titer neutralizing antibodies, and maintained high-levels of neutralizing antibodies [50]. In our study, both S and M segment genomes were screened for potential candidate vaccine T-cell epitopes that are conserved across the five genotypes. Based on the three scoring functions in the epitope presentation and their antigenicity, one epitope resulted in high scores predicted from the S segment. Multiple epitopes were generated from the M segment by the software program used. However, in the preponderance of the identified epitopes, proteasomal cleavage was lacking at the flanking regions and the epitopes were digested. Only one epitope survived the cleavage, but this epitope had low TAP score.

The studies on Hantavirus infections are limited from India but the problem is established to be significant in the Far East, Eastern Russia and South Korea. The present study was a bioinformatic approach. It would be necessary to demonstrate immune response to this epitope by in vitro experiments on cultured T cells from individuals exposed to Hantaviruses.

## Conclusion

The epitope MRNTIMASK from the nucleocapsid protein may be considered the most favorable moiety for the development of synthetic peptide vaccines. We propose that N protein derived Tcell epitopes may be suitable moieties for the development of subunit peptide vaccine.

## Figures and Tables

**Table 1:** List of epitopes from S and M segment that are present in all five genotypes and their prediction scores for suitability as potential peptide vaccine.

Epitopes	Peptide position	HLA supertypes	Proteasomal cleavage	TAP score	Vaxijen score
S segment					
YLTSFVVPI	125	HLA-A*02:01	X	4.947 (intermediate)	-0.1023 (non-antigen)
SYLRRTQSM	364	HLA-A*24:02; HLA-B*08:01;	X	4.015 (intermediate)	0.6938 (antigen)
MRNTIMASK	338	HLA-B*27:05	0.5044	6.211 (high)	0.7426 (antigen)
KSSFYQSYL	358	HLA-B*58:01	X	6.344 (high)	-0.1575 (non-antigen)
M segment					
HTDLELDFS	668	HLA-A*01:01	X	-0.227 (low)	2.1801 (antigen)
YTITSLFSL	452	HLA-A*02:01; HLA-A*26:01; HLA-B*39:01	X	3.954 (intermediate)	0.3892 (non-antigen)
EAFSEGGIF	388	HLA-A*26:01	X	8.597 (high)	0.1765 (non-antigen)
YRTLNLFRY	613	HLA-B*27:05	X	3.841 (intermediate)	-0.1871 (non-antigen)
WGSGVGFTL	981	HLA-B*39:01	0.5006	0.581(low)	0.6654 (antigen)
LCVPGFHGW	472	HLA-B*58:01	X	4.795 (intermediate)	-0.1392 (non-antigen)

X- indicates cleavage within the peptide
